# EUS Morphology Is Reliable in Selecting Patients with Mucinous Pancreatic Cyst(s) Most Likely to Benefit from Surgical Resection

**DOI:** 10.1155/2017/9863952

**Published:** 2017-09-07

**Authors:** Siddharth Javia, Satish Munigala, Sushovan Guha, Banke Agarwal

**Affiliations:** ^1^Division of Gastroenterology and Hepatology, Saint Louis University School of Medicine, Saint Louis, MO, USA; ^2^Saint Louis University Center for Outcomes Research, Saint Louis University, Saint Louis, MO, USA; ^3^Division of Gastroenterology, Hepatology and Nutrition, The University of Texas Medical School at Houston, Houston, TX, USA

## Abstract

**Background and Study Aims:**

Endoscopic ultrasound (EUS) surveillance of patients with mucinous pancreatic cysts relies on the assessment of morphologic features suggestive of malignant transformation. These criteria were derived from the evaluation of surgical pathology in patients with pancreatic cysts who underwent surgery. Reliability of these criteria when evaluated by EUS in identifying lesions which require surgery has still not been established.

**Patients and Methods:**

This retrospective cohort study included seventy-eight patients who underwent surgical resection of pancreatic cysts based on EUS-FNA (fine-needle aspiration) findings suggestive of mucinous pancreatic cysts with concern for malignancy.

**Results:**

Final surgical pathology diagnoses of patients were the following: adenocarcinoma (19), intraductal papillary mucinous neoplasm (IPMN) (39), mucinous cystic neoplasm (MCN) (13), serous cystadenoma (2), pseudocyst (3), mucinous solid-cystic lesion of indeterminate type (1), and mesenteric cyst (1). Cysts with focal wall thickening ≥ 3 mm (*p* = 0.0008), dilation of pancreatic duct (PD) (*p* = 0.0067), and cyst size ≥ 3 cm (*p* = 0.016) had significantly higher risk of adenocarcinoma. None of the patients without any of these morphologic features had cancer.

**Conclusions:**

In patients with mucinous pancreatic cyst(s), focal wall thickening, cyst size ≥ 3 cm, and PD dilation as assessed by EUS can help identify advanced mucinous cysts which require surgery and should routinely be evaluated during EUS surveillance.

## 1. Introduction

Mucinous pancreatic cysts should be considered for surgical resection due to their malignant potential [1]. However, pancreatectomy is associated with significant morbidity and mortality even in high volume centers [2–4]. Surgical resection is therefore recommended only in a subset of patients where the potential benefits seem to outweigh the risks of surgery [5]. Patients with benign appearing mucinous cysts and those who opt not to have surgery are usually placed in surveillance programs with follow-up imaging, preferably endoscopic ultrasound (EUS) performed at specified time intervals [6–8]. Ideally in patients undergoing surveillance, the mucinous pancreatic cyst should be resected before it turns malignant. Since fine-needle aspiration (FNA) cytology has a poor sensitivity in diagnosing premalignant and malignant mucinous cysts, it has limited value in surveillance to help determine if and when surgical resection is needed [9–11]. The EUS surveillance of pancreatic cysts is largely based on the evaluation of morphologic features of the cyst for evidence of cyst progression or malignant transformation. The commonly used EUS morphologic criteria for surveillance include cyst size, pancreatic duct (PD) dilation, and intramural nodules [5, 12, 13]. They were derived from review of surgical pathology in patients who underwent resection of pancreatic cysts. However, the utility of these morphologic features as assessed by EUS in identifying patients who will benefit from surgery and thereby in guiding further management of the cyst has still not been established.

The present study included patients preoperatively diagnosed to have mucinous cystic lesion (both side branch-intraductal papillary mucinous neoplasms (SB-IPMN) and mucinous cystic neoplasms (MCNs)) and referred to surgery based on EUS findings. We correlated EUS morphologic findings with surgical pathology and then determined which findings were most helpful in identifying mucinous cysts that will benefit from surgical resection.

## 2. Materials and Methods

### 2.1. Study Design and Participants

**Figure 1:**
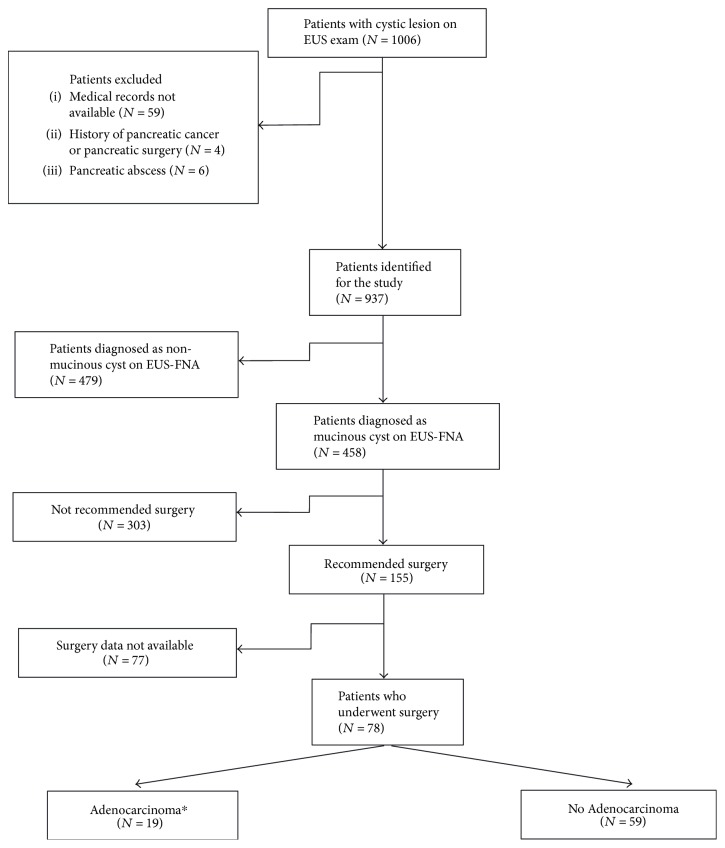
Study cohort. Mucinous cyst criteria: (1) aspiration of thick mucus from the cyst, (2) CEA > 192 *μ*g/ml, or (3) presence of mucinous epithelium in aspirate. Recommendation for surgery based on the presence of focal wall thickness, septa > 3 mm in thickness, cyst size ≥ 3 cm, cellular atypia, or adenocarcinoma on cytology or cyst fluid CEA > 500 ng/ml. Cysts with aspirates containing significant number of acute inflammatory cells were considered to be pseudocysts even if the aforementioned criteria were met. ^∗^Adenocarcinoma *N* = 19 included adenoca = 17 and IPMN high grade = 2. EUS-FNA: endoscopic ultrasound fine-needle aspiration; IPMN: intraductal papillary mucinous neoplasm.

This was a retrospective cohort study of patients with a cystic pancreatic lesion on EUS performed for the evaluation of a focal pancreatic lesion found on a CT scan/MRI/ultrasound between March 2002 and May 2013 at Saint Louis University Hospital and Missouri Baptist Hospital (*n* = 1006). Out of 1006 patients identified, the following patients were excluded: those with partially missing/unavailable medical records (*n* = 59) and those with a history of pancreatic cancer or pancreatic surgery (*n* = 4) and pancreatic abscess (*n* = 6) (Figure 1). Patients with prior history of pancreatic cancer or pancreatic surgery were excluded to obtain a clean cohort. This was to obviate any bias due to possible higher predisposition for dysplasia in the cyst in patients with pancreatic cancer or any other lesion that prompted previous pancreatic surgery. After exclusions, 937 patients were selected. 458 cases were diagnosed as mucinous cyst based on EUS-FNA. Criteria for the diagnosis as mucinous cyst included the following: (1) aspiration of thick mucus from the cyst, (2) CEA > 192 *μ*g/ml, or (3) presence of mucinous epithelium in aspirate. Cysts with aspirates containing a significant number of acute inflammatory cells were considered to be pseudocysts even if the aforementioned criteria were met. Surgery was recommended in 155 patients based on the presence of focal wall thickness > 3 mm, septa >3 mm in thickness, cyst size ≥3 cm, cellular atypia or adenocarcinoma on cytology, or cyst fluid CEA > 500 ng/ml. Of note, PD dilation was not a criterion for surgical referral. In our practice, we do not routinely look for communication of the cyst with PD and, therefore, this information is not available. Surgical operative notes and surgical pathology reports were available for 78 patients who were included in the final analysis. Remaining patients (*n* = 77) had no surgery-related data available because either they had declined surgery, were advised not to have surgery for various reasons, or were lost to follow up or their surgical notes/surgical pathology was not accessible.

Medical records of all the patients who were referred for surgery (*n* = 155) were reviewed for their age at the time of surgery; presence of symptoms like weight loss, jaundice, and history of acute pancreatitis; EUS characteristics like cyst size ≥ 3 cm, focal wall thickness (≥3 mm), and thickened septa (≥3 mm); and dilation of PD. PD was considered dilated if it was more than 3 mm, 2 mm, and 1 mm in the head, body, and tail of the pancreas, respectively. Final diagnosis was based on surgical pathology.

### 2.2. Statistical Analysis

Demographic characteristics, EUS findings, and patient symptoms were tested for significance for their association with a final diagnosis of mucinous adenocarcinoma using univariate analysis. Chi-square test was used for categorical variables, and *t*-test for age. Factors that appeared significant on univariate analysis were also tested using multivariate logistic regression analysis to see if these EUS characteristics were independent determinants of borderline or malignant stage of the mucinous cyst. All analyses were conducted using SAS version 9.2 (SAS Inc., Cary, North Carolina). Statistical significance was set at *P* value < 0.05. This study was approved by Missouri Baptist Medical Center Institutional Review Board (protocol number 994) and Saint Louis University Institutional Review Board (protocol number 23541).

## 3. Results

### 3.1. Patients Referred for Surgery

We compared the characteristics of 78 patients who were included for analysis and the remaining 77 patients who had no surgery data (Table 1). The proportion of patients with cyst size (≥3 mm), thickened septa, and adenocarcinoma/atypia on cytology is similar in these two groups.

### 3.2. Diagnosis Based on Surgical Pathology in Study Patients

Table 2 summarizes the final diagnosis based on surgical pathology amongst 78 patients included for the analysis. Only 72 of 78 patients were finally found to have mucinous cysts (positive predictive value = 92.3%; 95% CI (86.4, 98.2)). There were 4 false-positive diagnoses of malignancy based on FNA cytology. Surgical pathology in these patients revealed low grade IPMN (*n* = 2), pseudocyst (*n* = 1), and a mucinous solid-cystic lesion of indeterminate type (*n* = 1). There were three false-negative diagnosis by FNA cytology. Preoperative EUS-FNA cytology in these patients revealed mild epithelial atypia (*n* = 1), moderate dysplasia (*n* = 1), and cystic change without atypia (*n* = 1).

### 3.3. Patient Characteristics and EUS Findings in Patients with and without Adenocarcinoma

All patients included in this study were preoperatively diagnosed to have mucinous cystic lesions based on EUS-FNA. The EUS-FNA cytology was diagnostic of adenocarcinoma in 20 patients, benign in 39 patients, and atypical in 19 patients.  Table 3 summarizes the patient characteristics and EUS findings in all 78 patients included for the analysis and based on the final diagnosis. Patient age and symptoms including abdominal pain, weight loss, jaundice, or history of acute pancreatitis were not significantly associated with adenocarcinoma in this cohort. Focal wall thickening (*p* = 0.008), cyst size ≥ 3 cm (*p* = 0.016), and diffuse dilation of PD (*p* = 0.0067) but not septal thickening on EUS were significant predictors of adenocarcinoma. On multivariate logistic regression analysis, cyst size ≥ 3 cm and focal wall thickening were found to be significant independent predictors of adenocarcinoma but not PD dilation (Table 4).

### 3.4. Correlation of EUS Criteria with Cystic Lesions Requiring Surgery (CLRS)

**Figure 2:**
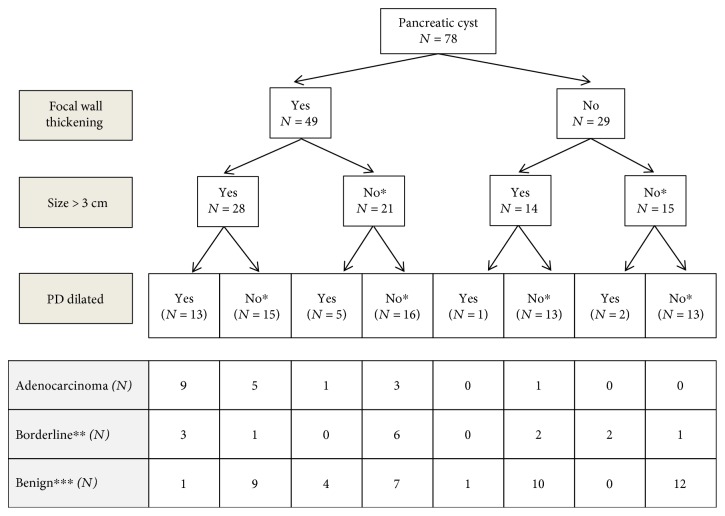
Correlation of EUS findings of focal wall thickening, cyst size ≥ 3 cm, and PD dilation with malignant, borderline, and benign pancreatic cysts. ^∗^No = either absent or missing. ^∗∗^Borderline category includes mucinous cysts with intermediate grade dysplasia. ^∗∗∗^Benign category includes benign mucinous as well as nonmucinous cysts. EUS: endoscopic ultrasound; PD: pancreatic duct.

**Figure 3:**
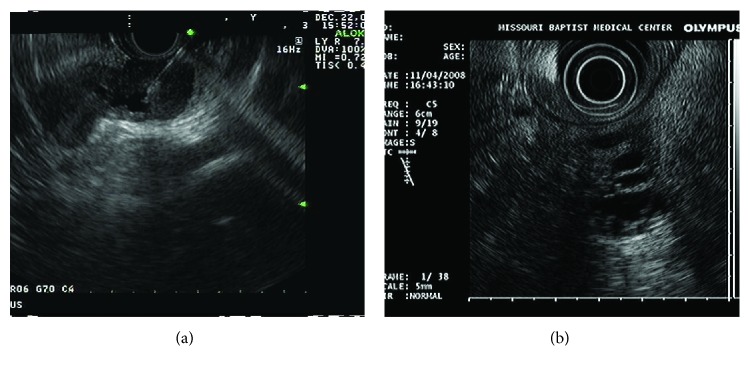
Endoscopic ultrasound imaging in a patient presenting with mucinous cystic lesion showing focal wall thickening.

Figure 2 summarizes how EUS morphologic finding of focal wall thickness, size ≥ 3 cm, and dilated PD correlates with the diagnosis of borderline and malignant cystic neoplasm on surgical pathology in study patients. Focal wall thickening (Figure 3) was the most helpful finding with 28 of 49 patients (57%) with this finding having lesions requiring surgery (malignant and borderline neoplasms) compared to 6 of 29 patients (21%) without it. Cyst size ≥ 3 cm in patients with focal wall thickening was associated with higher likelihood of lesions requiring surgery (14 malignant and 4 borderline lesions amongst 28 patients) compared to those without focal wall thickening (1 malignant and 2 borderline amongst 14 patients). PD dilation was associated with higher likelihood of malignant and borderline lesions in lesions with focal wall thickening and size ≥3 cm but was less helpful in remaining patients.

## 4. Discussion

In this study, we performed a clinicopathologic correlation in patients who underwent surgery based on the preoperative EUS-FNA diagnosis of mucinous cystic lesions with suspicion of malignancy. Amongst 78 study patients, 34 had lesions that warranted surgery (19 cancer and 15 borderline lesions). We found no correlation between patient age and symptoms and the likelihood of adenocarcinoma. Focal wall thickening (≥3 mm), cyst size ≥ 3 cm, and dilated PD were associated with higher likelihood of adenocarcinoma on univariate analysis, but only focal wall thickening and size ≥ 3 cm were independent predictors of adenocarcinoma on multivariate analysis. Eighteen of 49 patients with focal wall thickening and one of 29 patients without identifiable focal wall thickening on EUS were found to have adenocarcinoma. Cyst size and PD dilation were however useful adjunctive findings. None of patients lacking all three of these findings was found to have adenocarcinoma in our cohort.

Surgery is considered appropriate in patients with malignant or borderline pancreatic mucinous cysts with high near- or medium-term risk of malignant transformation [14]. However, preoperative identification of these patients is a challenge [5]. Currently in most centers, patients are selected for surgery based on the presence of symptoms, cyst size, and mural nodule on EUS, MRI, or CT scans [1, 5]. Cytology of EUS-FNA specimen especially exfoliative cytology from the cyst aspirate is not considered reliable due to its suboptimal sensitivity [9–11]. Cytology and cyst fluid biochemistry are helpful in initial evaluation and characterization of the pancreatic cyst. In our experience, it is much less helpful for the surveillance of patients with mucinous pancreatic cyst since bacterial contamination and oozing of blood into the cyst with FNA can render subsequent analysis of cyst contents including cytology less reliable. As a result, in most centers, surveillance of mucinous cysts by EUS involves evaluation of cyst for morphologic features suggestive of cyst progression or malignant transformation. In view of their widespread use in the management of patients with pancreatic cyst(s), rigorous evaluation and analysis of these morphologic criteria are warranted.

Most published data on predictors of malignancy in pancreatic cysts is derived from patients from surgical databases [5, 15]. Though a few of these studies have tried to incorporate the results of preoperative imaging, these published data have inherent recruitment bias [16]. In these studies, MCNs were more likely to be larger than 3 cm in size, have mural nodules with dilation of main PD, and were associated with symptoms. The criteria used for recommending surgery in these patients are not clearly discernible and vary widely. The positive predictive value and negative predictive value of these predictors of malignancy cannot be determined from these studies because of their study design. Our cohort comprises patients who were actually referred to surgery based on these clinical and EUS features. Since the study included patients over a long period, the criteria for surgery included those such as cyst fluid CEA > 500 ng/ml which were considered indication for surgery for some time but are no longer viewed that way in most centers. We included those patients so as not to inject a selection bias in our cohort. By correlating final surgical pathology with findings that prompted surgical referral, the present data enables validation of these morphologic criteria as assessed on EUS for selecting patients for surgery at the time of initial evaluation and for surveillance.

Even though we used commonly accepted and rather conservative criteria to diagnose mucinous cysts, not all patients preoperatively diagnosed to have mucinous cysts actually had a mucinous lesion; false-positive diagnosis included patients with serous cystadenoma, pseudocyst, and mesenteric cyst and one patient with a solid-cystic lesion whose etiology could not be determined even on surgical pathology. These data illustrate the limits of criteria used currently for diagnosing a mucinous cyst—a mucinous cyst aspirate, high CEA levels in cyst fluid, and presence of mucinous epithelium. Since mucinous cysts are more sinister clinically than the other types of pancreatic cysts and require surgery or surveillance, we believe that it is better that a few lesions that closely mimic mucinous cysts preoperatively be diagnosed as mucinous cysts rather than a mucinous cyst being missed due to the use of more stringent criteria.

In the present cohort, pancreatic adenocarcinoma/HGD was diagnosed preoperatively by EUS-FNA in 16 of 19 patients. Conversely, of the 20 patients preoperatively diagnosed to have cystadenocarcinoma/HGD (not just atypia), 16 were confirmed based on surgical pathology. The sensitivity for diagnosing malignancy is much higher than has been reported earlier by several groups [17, 18]. Cizginer and colleagues [17] found that EUS morphology, cytology, and CEA had sensitivity of 45.8%, 37.5%, and 78.2% for diagnosis of malignant mucinous cyst. Donahue and colleagues [18] were able to correctly predict only 67% of the pathological diagnosis and found that EUS could not improve the ability to preoperatively differentiate benign versus malignant cysts significantly. We believe this is because the previous studies have only evaluated exfoliative cytology from the cyst aspirates. We, however, aggressively target the focal wall thickening with a FNA needle and have onsite assessment by an attending cytologist about not only the adequacy of the cytology specimen but also a determination of malignancy. We use exfoliative cyst fluid cytology only for cysts without focal wall thickening on EUS. While evaluating the FNA specimens from the thickened part of the cyst wall, our cytologists not only look for features diagnostic of malignancy but also look for three-dimensional heaping of the epithelial cells to suggest progression to borderline cystic neoplasm.

In our clinical practice, we perform FNA of the cyst only at the time of initial evaluation of the cyst. We repeat FNA of the cyst at the time of follow-up EUS only when there is identifiable focal wall thickening. The purpose of EUS surveillance of mucinous pancreatic cysts is to identify not only cysts with overt malignancy but also borderline cystic neoplasms, which have the potential to turn malignant in the near or medium term. We, therefore, recommend surgery for patients with pancreatic cystic lesions with abnormal EUS morphology (particularly those with focal wall thickening) even if the cytology is just atypical or nondiagnostic. We interpret the absence of malignant epithelial cells in these lesions as suggestive of a borderline cystic neoplasm and do not discount the possibility that HGD or carcinoma in situ may be present in another part of the cyst. An effective surveillance of mucinous cysts would identify these lesions at a stage when they are borderline and precancerous (and have nonmalignant cytology) rather than when they turn frankly malignant. Our data provides support that EUS morphology of the mucinous cysts can be helpful in identifying these lesions and selecting patients who would benefit most from surgical resection.

Our cohort of patients with mucinous pancreatic cysts included those with SB-IPMN and MCNs, and we did not make a distinction in their management as suggested by the Sendai criteria. This is in part because our cohort included patients whose diagnosis predated development and general acceptance of the Sendai criteria. As per these criteria, surgery is recommended for a patient with MCN which is different in view of the younger age and the cumulative risk associated with surveillance. There is considerable overlap between SB-IPMN and MCN in terms of the communication between the PD and the cyst, making it difficult to reliably distinguish them preoperatively. There is no data to suggest that the EUS morphologic criteria that suggest malignancy differ between the MCN and SB-IPMN.

The present study has limitations inherent to its retrospective design. However, it is based on the cohort of patients who were referred for surgery based on the preoperative EUS-FNA diagnosis. The patients used in the final analysis were derived from 937 patients with pancreatic cystic lesions evaluated in our clinical practice over 10 years. The subset of patients who were referred for surgery but were excluded for analysis was similar to that of those who were included (Table 1). Patients with pancreatic cysts that appeared benign morphologically, had no atypical cells on cytology, and did not meet criteria for surgical resection were followed clinically. The final determination of etiology and presence of malignancy in these cysts cannot be made with certainty due to the absence of surgical pathology in these patients and slow progression of these cysts making this determination clinically difficult and unreliable. There is, unfortunately, no way around this limitation, though it is reassuring to know that amongst the patients who did undergo surgery, none with cysts without all three findings of focal wall thickening, PD dilation, and size > 3 cm were found to have malignancy. We do not use commercially available DNA analysis on the cyst aspirates as its benefit is still not established and is definitely not the standard of care [5]. The present study included patients who underwent EUS by a single operator. While this allowed for consistency in interpretation of EUS findings, it also did not allow for factoring in the interobserver variability in the interpretation of these findings. The present data are derived from a high volume referral practice and may not reflect the results in smaller and lower volume practices.

To conclude, EUS morphologic findings including focal wall thickening, cyst size ≥ 3 cm, and PD dilation can help identify patients likely to have malignant or borderline mucinous pancreatic cysts. These findings are helpful for EUS surveillance in patients with mucinous pancreatic cyst(s) and are useful adjunct to cytology in the determination of malignancy in a pancreatic cyst.

## Figures and Tables

**Table 1:** Comparison between patients with surgery data available and patients who did not have surgery or surgery data not available.

Indication for surgery	Patients who had surgery data *N* = 78	Patients who did not have surgery or no surgery data available *N* = 77
Size ≥ 3 cm	42 (53.8%)	45 (58.4%)
Focal wall thickness	49 (62.8%)	33 (42.8%)
Thickened septa	25 (32.1%)	14 (18.2%)
Atypia/adenocarcinoma	39 (50%)	33(42.8%)
Atypia	19 (25.6%)	11 (14.3%)
Adenocarcinoma^∗^	20 (25.4%)	22 (28.6%)
CEA > 500	32 (41%)	11 (14.3%)
PD dilation	22(28.2%)	25(32.5%)
People with only 1 indication for surgery		
*N*	20 (25.6%)	40 (51.9%)
Size ≥ 3 cm	7 (9%)	21 (27.3%)
Focal wall thickness	1	4 (5.2%)
Thickened septa	0	1
Atypia/adenocarcinoma	3 (3.8%)	3 (3.9%)
CEA > 500	9 (11.5%)	11 (14.3%)

^∗^Adenocarcinoma includes pts with adenocarcinoma and high grade atypia noted on cytology. CEA: carcinoembryonic antigen; PD: pancreatic duct.

**Table 2:** Final diagnosis of surgically resected patients.

Patients selected	Total = 78 *N* (%)
Pseudocyst	3 (3.85%)
IPMN low grade	25 (32.05%)
IPMN intermediate grade	14 (17.95%)
MCN low grade	12 (15.38%)
MCN intermediate grade	1 (1.28%)
Mucinous solid-cystic lesion of indeterminate type	1 (1.28%)
Serous cyst	2 (2.56%)
Mesenteric cyst	1 (1.28%)
Adenocarcinoma	17 (21.79%)
IPMN high grade	2 (2.56%)

IPMN: intraductal papillary mucinous neoplasm; MCN: mucinous cystic neoplasm.

**Table 3:** Characteristics of patients who underwent surgery.

Patients selected (*n* = 78)	Adeno Ca Yes = 19 *N* (%)	Adeno Ca No = 59 *N* (%)	*P* value	Total = 78 *N* (%)
Gender				
Female	6 (31.57%)	36 (61.01%)	0.035	42 (53.9%)
Male	13 (68.43%)	23 (38.98%)		36 (46.1%)
Symptoms				
Abdominal pain	5 (26.31%)	19 (32.20%)	0.77	24 (30.8%)
Weight loss	6 (31.57%)	12 (20.33%)	0.35	18 (23.1%)
Jaundice	3 (15.78%)	2 (3.38%)	0.09	5 (6.4%)
Acute pancreatitis	3 (15.78%)	9 (15.25%)	1.00	12 (15.4%)
	*Mean (SD)*	*Mean (SD)*		*Mean (SD)*
Age (years)	66.82 (8.48)	65.27 (10.75)	0.56	65.6 (10.22)
*EUS features*	*Yes = 19* *N (%)*	*No = 59* *N (%)*	*P value*	Total = 78 *N* (%)
Cyst size ≥ 3 cm	15 (78.94%)	27 (45.76%)	0.016	42 (53.8%)
Thickened septa	8 (42.10%)	17 (28.81%)	0.39	25 (32.1%)
Focally thickened cyst wall	18 (94.73%)	31 (52.54%)	0.0008	49 (62.8%)
Dilated PD	10 (52.63%)	11 (18.64%)	0.0067	21 (26.9%)
Atypia/adenocarcinoma on cytology	18^∗^ (94.73%)	21^∗∗^ (35.59%)	0.0001	39 (50.0%)

^∗^18 patients include 16 patients with adenocarcinoma and 2 patients with atypia on cytology. ^∗∗^21 patients include 4 patients with adenocarcinoma and 17 patients with atypia on cytology. Adeno Ca: adenocarcinoma; SD: standard deviation; EUS: endoscopic ultrasound; PD: pancreatic duct.

**Table 4:** Logistic regression of adenocarcinoma.

Patients selected (*n* = 78)	Odds ratio	95% CI	*P* value
Adenocarcinoma			
Thick cyst wall	12.59	1.49–106.43	0.020
Size ≥ 3 cm	4.02	1.07–15.00	0.038
Dilated PD	2.68	0.76–9.33	0.12

Adenocarcinoma includes pts with adenocarcinoma and high grade atypia noted on cytology. PD: pancreatic duct; CI: confidence interval.

## Abbreviations

CEA: Carcinoembyronic antigen
EUS: Endoscopic ultrasound
FNA: Fine-needle aspiration
PD: Pancreatic duct
MCN: Mucinous cystic neoplasm
IPMN: Intraductal papillary mucinous neoplasm
SD: Standard deviation.
